# Pterostilbene Enhances Cytotoxicity and Chemosensitivity in Human Pancreatic Cancer Cells

**DOI:** 10.3390/biom10050709

**Published:** 2020-05-04

**Authors:** Yi-Hao Hsu, Sheng-Yi Chen, Sheng-Yang Wang, Jer-An Lin, Gow-Chin Yen

**Affiliations:** 1 Department of Food Science and Biotechnology, National Chung Hsing University, 145 Xingda Road, Taichung 40227, Taiwan; alston0708@gmail.com (Y.-H.H.); wilson1211983@gmail.com (S.-Y.C.); 2 Department of Forestry, National Chung Hsing University, 145 Xingda Road, Taichung 40227, Taiwan; taiwanfir@dragon.nchu.edu.tw; 3 Graduate Institute of Food Safety, National Chung Hsing University, 145 Xingda Road, Taichung 40227, Taiwan; lja@dragon.nchu.edu.tw

**Keywords:** apoptosis, autophagy, chemosensitivity, gemcitabine, pancreatic ductal adenocarcinoma, pterostilbene, MDR1

## Abstract

Gemcitabine (GEM) drug resistance causes high mortality rates and poor outcomes in pancreatic ductal adenocarcinoma (PDAC) patients. Receptor for advanced glycation end products (RAGE) involvement in the GEM resistance process has been demonstrated. Therefore, finding a safe and effective way to inhibit receptors for RAGE-initiated GEM resistance is urgent. Pterostilbene (PTE), a natural methoxylated analogue derived from resveratrol and found in grapes and blueberries, has diverse bioactivities, such as antioxidative, anti-inflammatory, and anticancer qualities. The overall research objective was to determine the potential of PTE to enhance tumor cytotoxicity and chemosensitivity in PDAC cells. Our results have demonstrated that PTE induced S-phase cell cycle arrest, apoptosis, and autophagic cell death and inhibited multidrug resistance protein 1 (MDR1) expression by downregulating RAGE/PI3K/Akt signaling in both MIA PaCa-2 and MIA PaCa-2 ^GEMR^ cells (GEM-resistant cells). Remarkably, convincing evidence was established by RAGE small interfering RNA transfection. Taken together, our study demonstrated that PTE promoted chemosensitivity by inhibiting cell proliferation and MDR1 expression via the RAGE/PI3K/Akt axis in PDAC cells. The observations in these experiments indicate that PTE may play a crucial role in MDR1 modulation for PDAC treatment.

## 1. Introduction

Over 90% of pancreatic ductal adenocarcinomas (PDACs) are exocrine pancreatic cancer, which is a highly malignant tumor that with the growing cause of cancer-related mortality worldwide. In the early stages of PDAC, the signs or symptoms are rarely noticeable, and patients often have local invasion or distant metastasis at diagnosis. Unfortunately, less than 15%–20% of stage I-II PDAC patients are resectable due to most PDAC patients have carcinoma in-situ intravasation, perineural invasion, and distant metastasis at diagnosis [1]. The poor prognosis and outcomes of PDAC patients lead to a low five-year survival rate (6%) and a high incidence [2]. A report by the American Association for Cancer Research (AACR) estimated that PDAC may grow into the second leading cause of cancer-associated deaths by 2030 [3]. Thus, PDAC is a silent disease that remains a therapeutic challenge.

Gemcitabine (GEM), a deoxycytidine nucleoside analogue for cancer DNA synthesis obstruction, is the most common chemotherapeutic drug for PDAC treatment. However, approximately 75% of PDAC patients have GEM resistance, which greatly contributes to treatment failure [4]. In addition, the overall survival rate of patients treated with GEM chemotherapy is not significantly different between patients with local and distant recurrence, indicating that an effective prevention strategy is urgently required [5,6].

Pterostilbene (PTE), a natural methoxylated analogue, is derived from resveratrol, which is produced by Pterocarpus plants, grapes, and blueberries. A previous report showed that PTE has diverse bioactivities, including antioxidant, anti-inflammatory, and anticancer activities [7]. PTE treatment effectively suppresses the growth of several tumor cells, such as urinary bladder, colon, lung, prostate, breast, gastric cancers, and leukemia, through autophagy and apoptosis induction [8,9,10,11,12,13,14]. In addition, PTE triggers autophagy and apoptotic cell death and consequently inhibits multidrug resistance protein 1 (MDR1) expression, which has been observed in leukemia and lung cancer, indicating that PTE may strengthen the efficiency of chemotherapy [8,15]. However, the role of PTE in PDAC chemotherapy enhancement remains unknown.

Moreover, receptor for advanced glycation end products (RAGE) is highly expressed in pancreatic tumor tissue relative to normal adjacent tissue [16]. Recently, we found that RAGE enhances MDR1 expression in a GEM-resistant pancreatic carcinoma cell line (designated as MIA PaCa-2 ^GEMR^) [17]. Notably, quercetin treatment or RAGE silencing significantly inhibits RAGE-initiated PDAC progression and drug resistance by promoting autophagy and apoptosis [17]. PTE, a compound structurally related to quercetin, has been shown to have an inhibitory effect on RAGE-induced oxidative damage and inflammation in RAW 264.7 cells [18,19]. Accordingly, our objective in this study was to uncover the role of PTE in PDAC chemotherapy enhancement in the underlying RAGE-associated mechanism.

## 2. Materials and Methods

### 2.1. Chemicals and Reagents

DMEM, horse serum, and Opti-MEM were bought from Thermo Fisher Scientific (Waltham, MA, USA). FBS was purchased from NEQQ International Biological Corporation (Hong Kong, China). RIPA lysis buffer, ECL substrates, and polyvinylidene difluoride (PVDF) membranes were obtained from Millipore (Billerica, MA, USA). The PTE was kindly provided by professor Chi-Tang Ho (Rutgers University, USA). The GEM was purchased from Sigma-Aldrich (St. Louis, MO, USA). β-actin, beclin-1, and autophagy gene 5 (ATG5) antibodies were obtained from Novus Biologicals (Littleton, CO, USA). Bcl2-associated x protein (Bax), B-cell lymphoma-extra large (Bcl-xL), PI3K, Phospho-PI3K, Akt, Phospho-Akt, and MDR1 antibodies were bought from Cell signaling technology (Danvers, MA, USA). RAGE antibody was obtained from Invitrogen (Waltham, MA, USA). Microtubule-associated protein light chain 3 II (LC3 II) antibody, peroxidase goat anti-mouse and peroxidase goat anti-rabbit were purchased from GeneTex (Irving, CA, USA). RAGE siRNA and Dharmacon transfection reagent were purchased from GE Healthcare (Lafayette, CO, USA).

### 2.2. Cell Culture

MIA PaCa-2 cells (BCRC NO. 60139) were obtained from the Bioresource Collection and Research Center (HsinChu, Taiwan). A stable GEM-resistant PDAC cell line (denoted MIA PaCa-2 ^GEMR^) was established as reported previously [17]. Cells were maintained according to the American Type Culture Collection (ATCC) guidelines.

### 2.3. Cell Viability Analysis

Approximately 2 × 10^4^ cells/well were seeded into 96-well plates and cultured overnight. After cell treatment with PTE (0, 5, 10, 25, 50, and 75 μM) for 48 or 72 h, an MTT-based viability assay was performed as reported previously [17].

### 2.4. Cell Cycle Measurement

The cells were incubated with different concentrations of PTE for 72 h. The cell cycle was measured by propidium iodide (PI) staining and analyzed by FACScan flow cytometry (BD Biosciences, Franklin Lakes, NJ, USA) [17].

### 2.5. RAGE siRNA Transfection

The procedure was performed as reported previously [17]. Briefly, cells at a density of 1 × 10^5^ cells/mL were cultured on 6-well plates overnight and then incubated with 25 nM non-targeting control siRNA or RAGE siRNA for 24 h. The knockdown efficiency was verified by western blot analysis.

### 2.6. Western Blot Analysis

Western blotting was performed as reported previously [17]. Bcl2-associated x protein (Bax), B-cell lymphoma-extra large (Bcl-xL), PI3K, phospho-PI3K, Akt, phospho-Akt, beclin-1, and autophagy gene 5 (ATG5), microtubule-associated protein light chain 3 II (LC3 II) and MDR1 protein expression were evaluated. The relative intensities were measured by a Biospectrum 810 AC Imaging System (UVP, Upland, CA, USA) and normalized to the β-actin control.

### 2.7. Statistical Analysis

Experimental results were repeated in three independent operations and quantified as the mean ± standard deviation (SD). Data were analyzed by Student’s *t* test using SPSS 16.0 statistical software (IBM Corporation, Armonk, NY, USA).

## 3. Results

### 3.1. PTE Induced S-Phase Cell Cycle Arrest in PDAC Cell Lines

**Figure 1:**
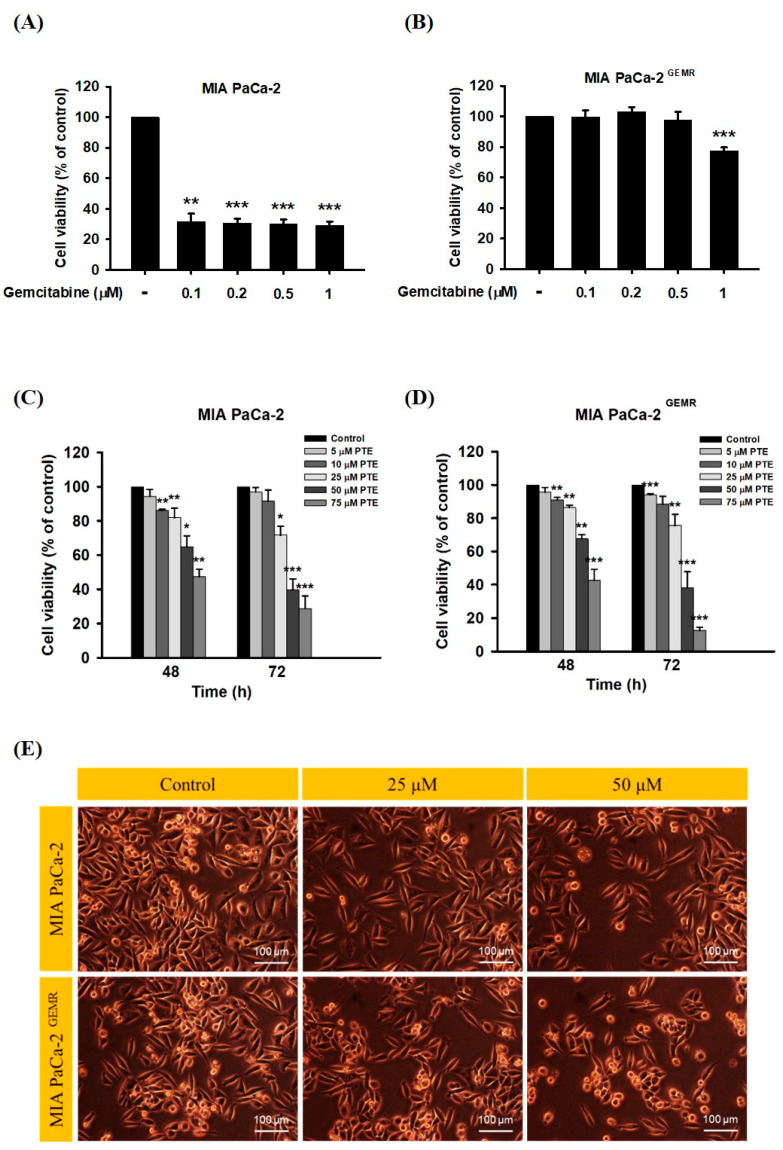
Effect of gemcitabine on cell viability and morphology in MIA PaCa-2 and MIA PaCa-2 ^GEMR^ cells. (**A**) MIA PaCa-2 and (**B**) MIA PaCa-2 ^GEMR^ cells were treated with different doses of gemcitabine for 72 h, and cell viability was analyzed by MTT assay. (**C**) MIA PaCa-2 and (**D**) MIA PaCa-2 ^GEMR^ cells were treated with different doses of pterostilbene for 48 and 72 h, and the cell viability was analyzed by MTT assay. (**E**) Representative phase-contrast images of MIA PaCa-2 and MIA PaCa-2 ^GEMR^ cells after treatment with 25 and 50 μM pterostilbene for 72 h. The results are shown as the mean ± SD (*n* = 3). *p* values were considered statistically significant when *p < 0.05, **p < 0.01, and ***p < 0.001 compared with the untreated control.

**Figure 2:**
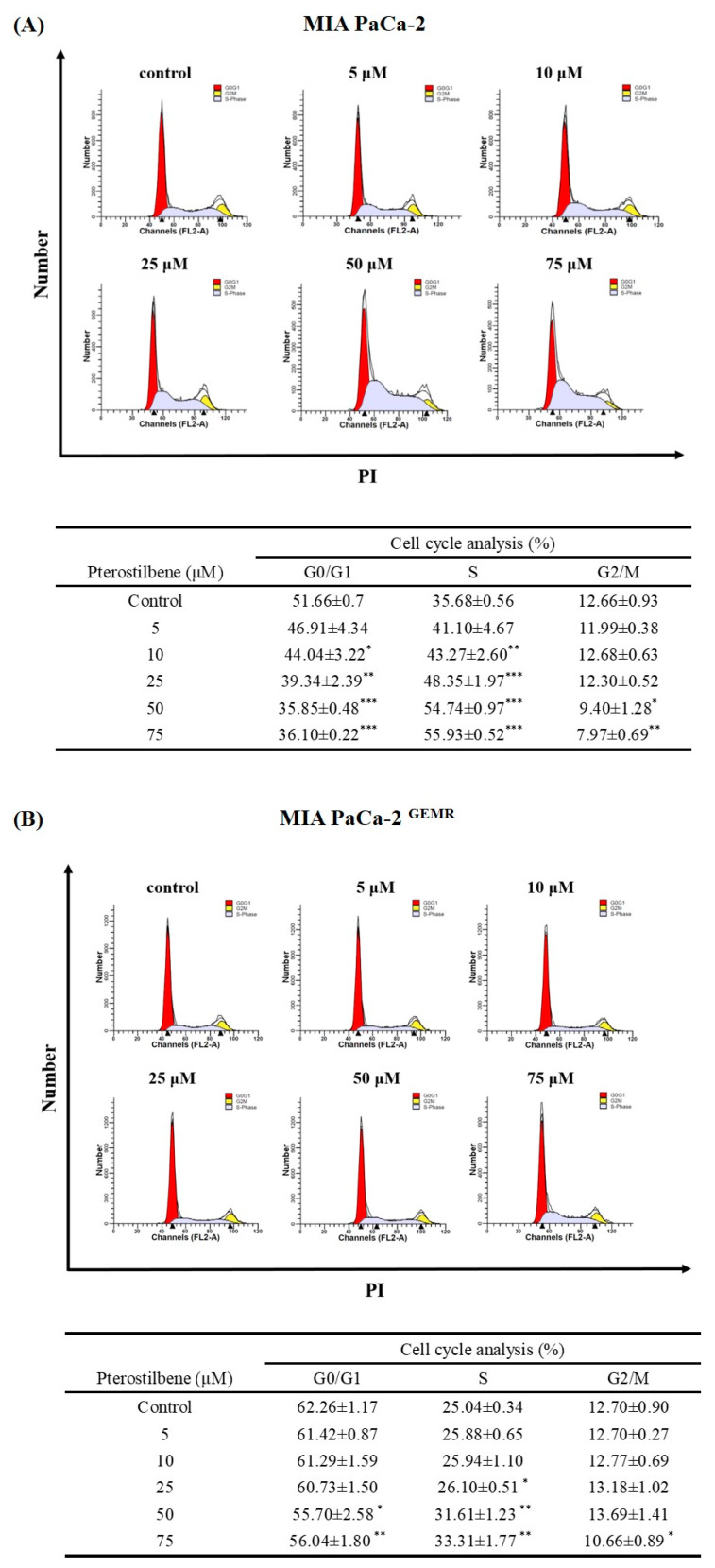
Effect of pterostilbene on the cell cycle of MIA PaCa-2 cells and MIA PaCa-2 ^GEMR^ cells. (**A**) MIA PaCa-2 and (**B**) MIA PaCa-2 ^GEMR^ cells were treated with 0–75 μM pterostilbene for 72 h, and PI staining was used to evaluate the cell cycle. The proportion of cells in each phase of the cell cycle is expressed as the mean ± SD (*n* = 3). *p* values were considered statistically significant when *p < 0.05, **p < 0.01, and ***p < 0.001 compared with the untreated control.

A stable GEM-tolerant MIA PaCa-2 ^GEMR^ cell line that can resist 0.5 μM GEM-induced cytotoxicity was established (Figure 1A,B). To assess the cytotoxicity effect triggered by PTE in both MIA PaCa-2 and MIA PaCa-2 ^GEMR^ cells. Cells were treated with PTE at different concentrations (0, 5, 10, 25, 50, and 75 μM) for 48 or 72 h. Cell proliferation suppressed by PTE treatment in a time- and dose-response manner was observed by MTT analysis (Figure 1C, D). The IC_50_ values of PTE in MIA PaCa-2 and MIA PaCa-2 ^GEMR^ cells were 41.8 and 42.0 μM (72 h), respectively. The spindle-shaped morphology and loss of viability by PTE treatment for 72 h compared with untreated cells are shown in Figure 1E. In addition, the detailed role of PTE on cell proliferation was validated by cell cycle analysis. Cell cycle analysis with propidium iodide (PI) staining showed that S-phase arrest was induced in PTE-treated MIA PaCa-2 cells compared to untreated cells in a dose-dependent manner (Figure 2A). Similar results were observed in GEM-resistant cells (Figure 2B), indicating that cell proliferation inhibition was induced via PTE-induced S-phase cell cycle arrest in both cell types.

### 3.2. PTE Triggered Apoptotic and Autophagic Cell Death in PDAC Cell Lines

**Figure 3:**
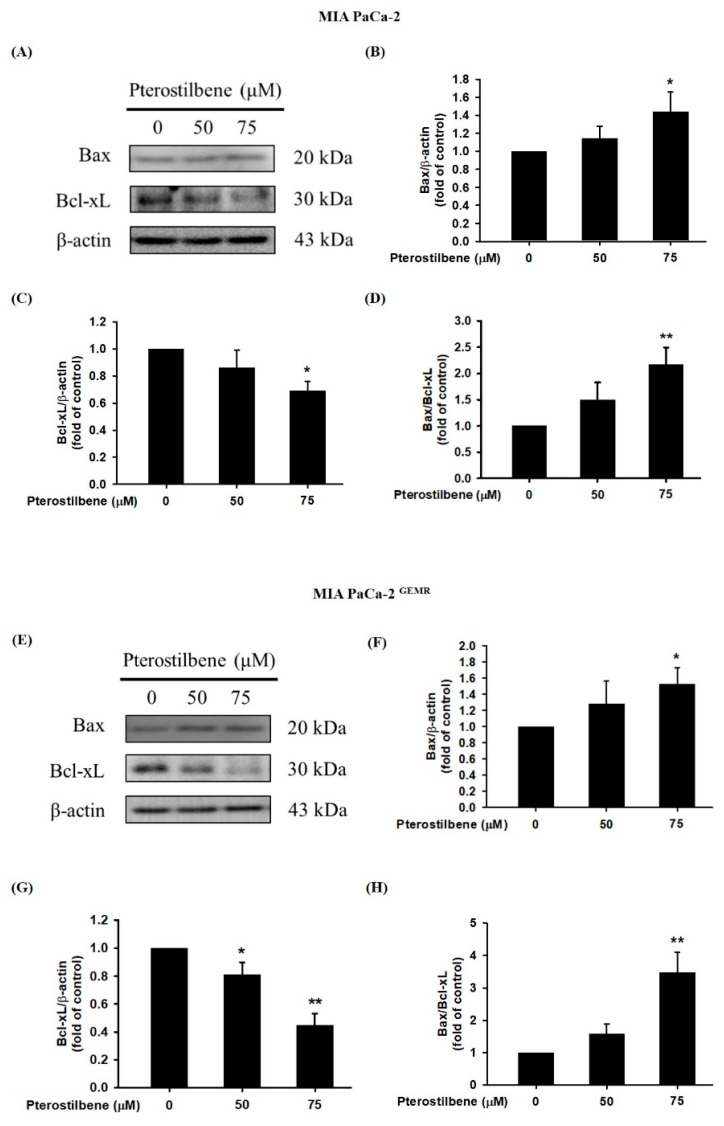
Effect of pterostilbene on apoptosis-related protein expression in MIA PaCa-2 cells and MIA PaCa-2 ^GEMR^ cells. Cells were treated with 50 and 75 μM pterostilbene for 72 h, and the expression levels of Bax and Bcl-xL protein in (**A**) MIA PaCa-2 and (**E**) MIA PaCa-2 ^GEMR^ cells were analyzed by western blotting. The relative expression levels of (**B**,**F**) Bax, (**C**,**G**) Bcl-xL, and (**D**,**H**) the ratio of Bax/Bcl-xL are expressed as the mean ± SD (*n* = 3). *p* values were considered statistically significant when *p < 0.05, **p < 0.01, and ***p < 0.001 compared with the untreated control.

**Figure 4:**
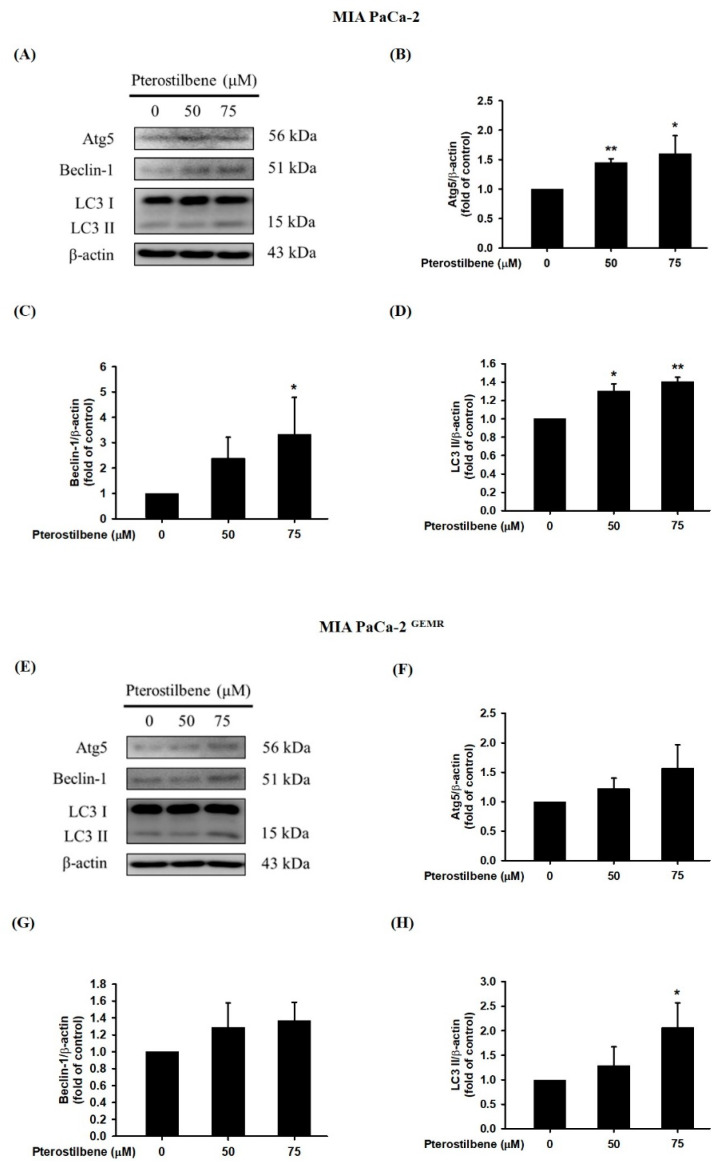
Effect of pterostilbene on autophagy-related protein expression in MIA PaCa-2 cells and MIA PaCa-2 ^GEMR^ cells. Cells were treated with 50 and 75 μM pterostilbene for 72 h, and the expression levels of Atg5, Beclin-1 and LC3 II protein in (**A**) MIA PaCa-2 and (**E**) MIA PaCa-2 ^GEMR^ cells were analyzed by western blotting. The relative expression levels of (**B**,**F**) Atg5, (**C**,**G**) Beclin-1, and (**D**,**H**) LC3 II are expressed as the mean ± SD (*n* = 3). *p* values were considered statistically significant when *p < 0.05, **p < 0.01, and ***p < 0.001 compared with the untreated control.

A recent report found that PTE induces cell cycle arrest-mediated apoptotic progression in ovarian cancer [20]. Regarding the anticancer characteristics of PTE, our study demonstrated that PTE treatment degraded Bcl-xL and elevated Bax protein expression in a dose-dependent manner in both cell lines (Figure 3). Our research also focused on PTE in autophagic cell death modulation. As shown in Figure 4A–D, PTE significantly enhanced Atg5, Beclin-1, and LC3-II protein expression in MIA PaCa-2 cells. Moreover, the protein levels of Atg5 and Beclin-1 were increased by PTE treatment in a dose-dependent manner but did not reach statistical significance in MIA PaCa-2 ^GEMR^ cells (Figure 4E–G). Nevertheless, LC3-II significantly increased in MIA PaCa-2 ^GEMR^ cells subjected to 75 μM PTE treatment compared to the untreated cells (Figure 4E,H). These results suggested that PTE induced apoptosis and autophagy in parental and GEM-resistant PDAC cells.

### 3.3. Autophagy Induction Was Required for PI3K/Akt Signaling Pathway Inhibition

**Figure 5:**
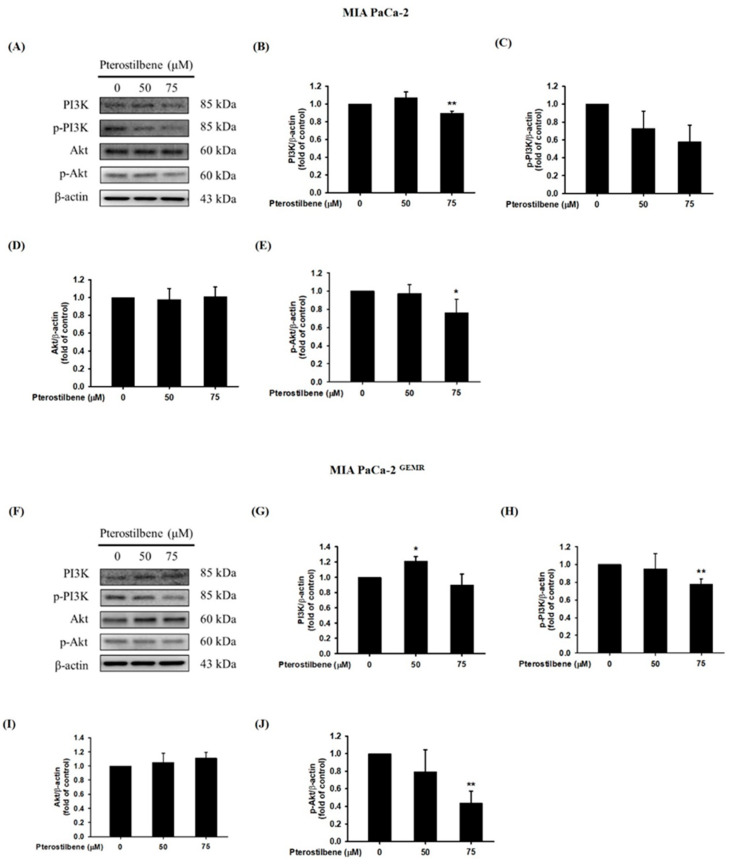
Effect of pterostilbene on PI3K/Akt protein expression in MIA PaCa-2 cells and MIA PaCa-2 ^GEMR^ cells. Cells were treated with 50 and 75 μM pterostilbene for 72 h, and the expression levels of PI3K and Akt protein in (**A**) MIA PaCa-2 and (**F**) MIA PaCa-2 ^GEMR^ cells were analyzed by western blotting. The relative expression levels of (**B**,**G**) PI3K, (**C**,**H**) p-PI3K, (**D**,**I**) Akt, and (**E**,**J**) p-Akt are expressed as the mean ± SD (*n* = 3). *p* values were considered statistically significant when *p < 0.05, **p < 0.01, and ***p < 0.001 compared with the untreated control.

Activation of the PI3K/Akt cascade is highly related to carcinogenesis, tumor progression, and chemoresistance. Targeting the PI3K/Akt signaling pathway is an ongoing effort in several clinical trials [21,22]. Currently, our evidence shows that quercetin treatment or RAGE silencing significantly increases apoptosis, autophagy, and chemosensitivity by suppressing the PI3K/Akt axis in PDAC [17]. Here, our results showed that 75 μM PTE treatment significantly inhibited the phosphorylation activities of PI3K and Akt proteins in both cell lines (Figure 5).

### 3.4. Higher RAGE and MDR1 Protein Levels Were Found in GEM-Resistant PDAC Cells

**Figure 6:**
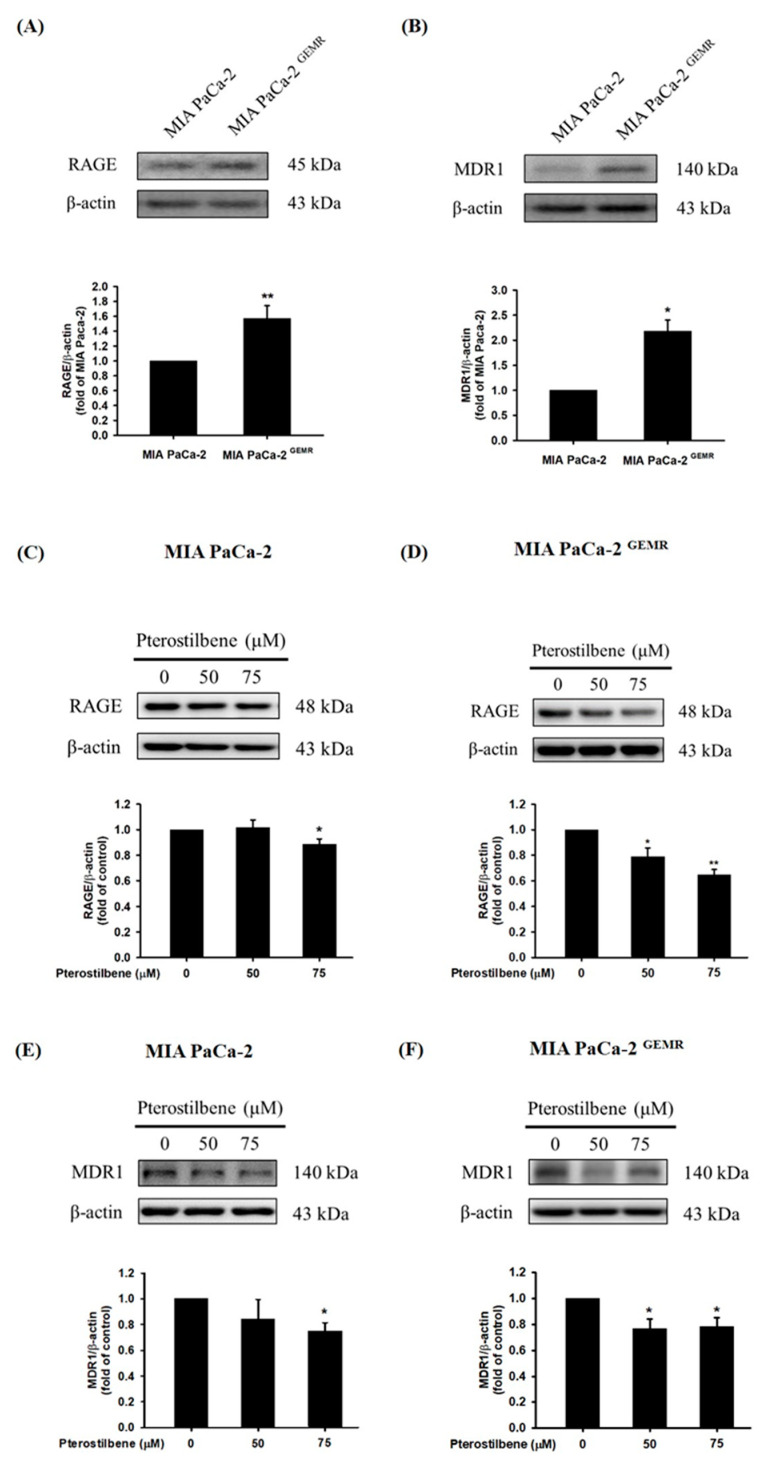
Effect of pterostilbene on RAGE and MDR1 protein expression in MIA PaCa-2 and MIA PaCa-2 ^GEMR^ cells. The expression levels of (**A**) RAGE and (**B**) MDR1 protein in MIA PaCa-2 and MIA PaCa-2 ^GEMR^ cells were analyzed by western blotting. RAGE protein expression in (**C**) MIA PaCa-2 and (**D**) MIA PaCa-2 ^GEMR^ cells was measured. MDR1 protein expression in (**E**) MIA PaCa-2 and (**F**) MIA PaCa-2 ^GEMR^ cells was measured. *p* values were considered statistically significant when *p < 0.05, **p < 0.01, and ***p < 0.001 compared with the untreated control. The results are expressed as the mean ± SD (*n* = 3).

High RAGE expression is correlated with chemotherapy resistance in PDAC patients [23,24]. Although the underlying molecular mechanism is still unclear, our current evidence demonstrates that abundant RAGE expression in MIA PaCa-2 ^GEMR^ cells consequently enhances GEM chemoresistance [17]. Similarly, higher RAGE protein expression was observed in GEM-resistant cells compared to MIA PaCa-2 cells in this study (Figure 6A). In addition, a high level of MDR1 protein expression was found in MIA PaCa-2 ^GEMR^ cells (Figure 6B). Apoptosis and autophagy are both key mediators in chemotherapy-induced cancer cell death [25,26]. RAGE and MDR1 protein expression, as well as cell proliferation were assessed to validate the effect of PTE on chemosensitivity in PDAC. RAGE expression was suppressed in PTE-treated MIA PaCa-2 cells compared to untreated cells (Figure 6C). In addition, PTE treatment significantly repressed RAGE protein levels in a dose-dependent manner in MIA PaCa-2 ^GEMR^ cells (Figure 6D). Interestingly, MDR1 protein expression was reduced by 75 μM PTE treatment in both cell lines (Figure 6E,F).

### 3.5. MDR1 Expression Was Upregulated by the RAGE-Initiated PI3K/Akt Signaling Pathway

**Figure 7:**
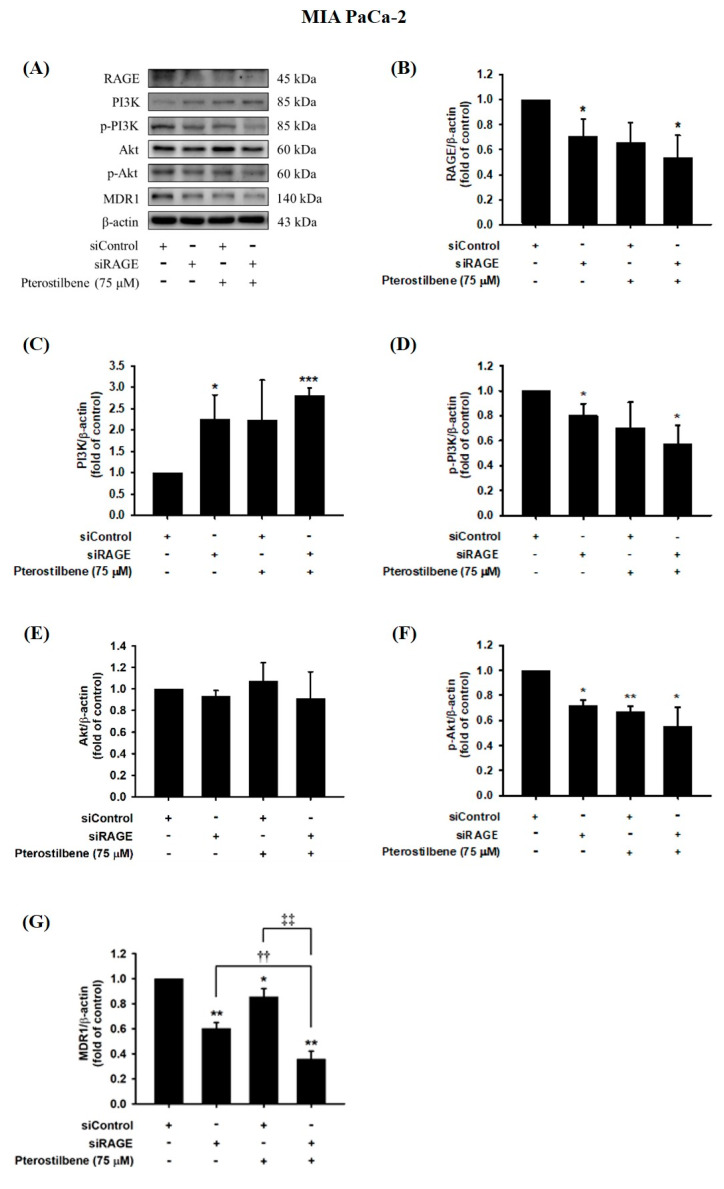
Effect of pterostilbene on RAGE, PI3K, Akt, and MDR1 expression in RAGE knockdown MIA PaCa-2 cells. Cells were transfected with 25 nM control siRNA or siRAGE for 24 h followed by 75 μM pterostilbene treatment for another 72 h. (**A**) The levels of MDR1, PI3K, Akt, and RAGE protein expression in RAGE knockdown MIA PaCa-2 cells were analyzed by western blotting. The relative expression levels of (**B**) RAGE, (**C**) PI3K, (**D**) p-PI3K, (**E**) Akt, (**F**) p-Akt, and (**G**) MDR1 in RAGE knockdown MIA PaCa-2 cells are expressed as the mean ± SD (*n* = 3). *p* values were considered statistically significant when *p < 0.05, **p < 0.01, and ***p < 0.001 compared with the untreated control. ††*p* < 0.01 and ‡‡*p* < 0.01 compared with pterostilbene-treated siRAGE cells.

**Figure 8:**
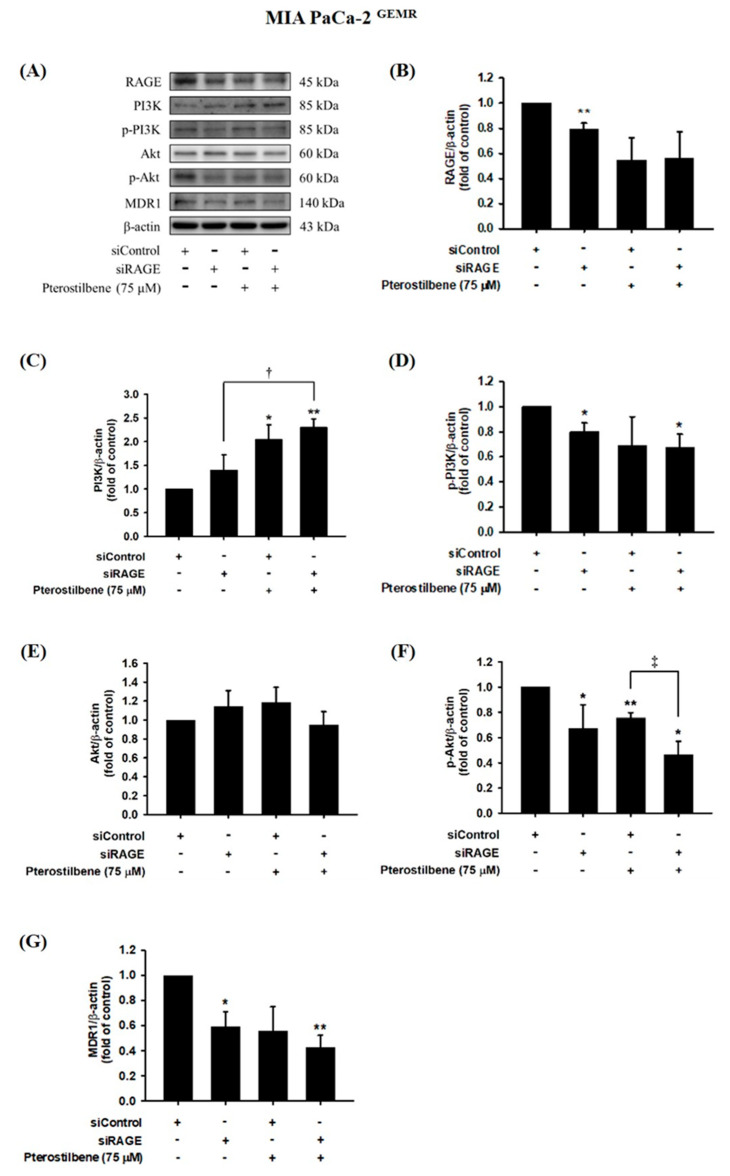
Effect of pterostilbene on RAGE, PI3K, Akt, and MDR1 expression in RAGE knockdown MIA PaCa-2 ^GEMR^ cells. Cells were transfected with 25 nM control siRNA or siRAGE for 24 h followed by 75 μM pterostilbene treatment for another 72 h. (**A**) The expression levels of MDR1, PI3K, Akt, and RAGE protein in RAGE knockdown MIA PaCa-2 ^GEMR^ cells were analyzed by western blotting. The relative expression levels of (**B**) RAGE, (**C**) PI3K, (**D**) p-PI3K, (**E**) Akt, (**F**) p-Akt, and (**G**) MDR1 in RAGE knockdown MIA PaCa-2 ^GEMR^ cells are expressed as the mean ± SD (*n* = 3). *p* values were considered statistically significant when *p < 0.05, **p < 0.01, and ***p < 0.001 compared with untreated control. †*p* < 0.05 and ‡*p* < 0.05 compared with pterostilbene-treated siRAGE cells.

We next examined whether MDR1 protein expression was regulated by the RAGE-initiated PI3K/Akt signaling pathway. Knocking down RAGE expression significantly reduced PI3K and Akt phosphorylation levels and consequently inhibited MDR1 expression in MIA PaCa-2 cells (Figure 7). In addition, decreases in RAGE, p-PI3K, p-Akt, and MDR1 protein levels were observed in PTE-treated siRAGE MIA PaCa-2 cells compared to siControl cells (Figure 7). Moreover, siRAGE or PTE treatment dramatically reduced MDR1 protein expression compared to that in the corresponding control cells (Figure 7G). In addition, suppression of PI3K and Akt protein phosphorylation levels and consequent inhibition of MDR1 expression was also observed in RAGE-silenced MIA PaCa-2 ^GEMR^ cells (Figure 8). Furthermore, PTE treatment significantly downregulated p-PI3K, p-Akt, and MDR1 protein levels in siRAGE MIA PaCa-2 ^GEMR^ cells compared with siControl cells (Figure 8).

### 3.6. Chemosensitivity Induced by PTE Treatment

**Figure 9:**
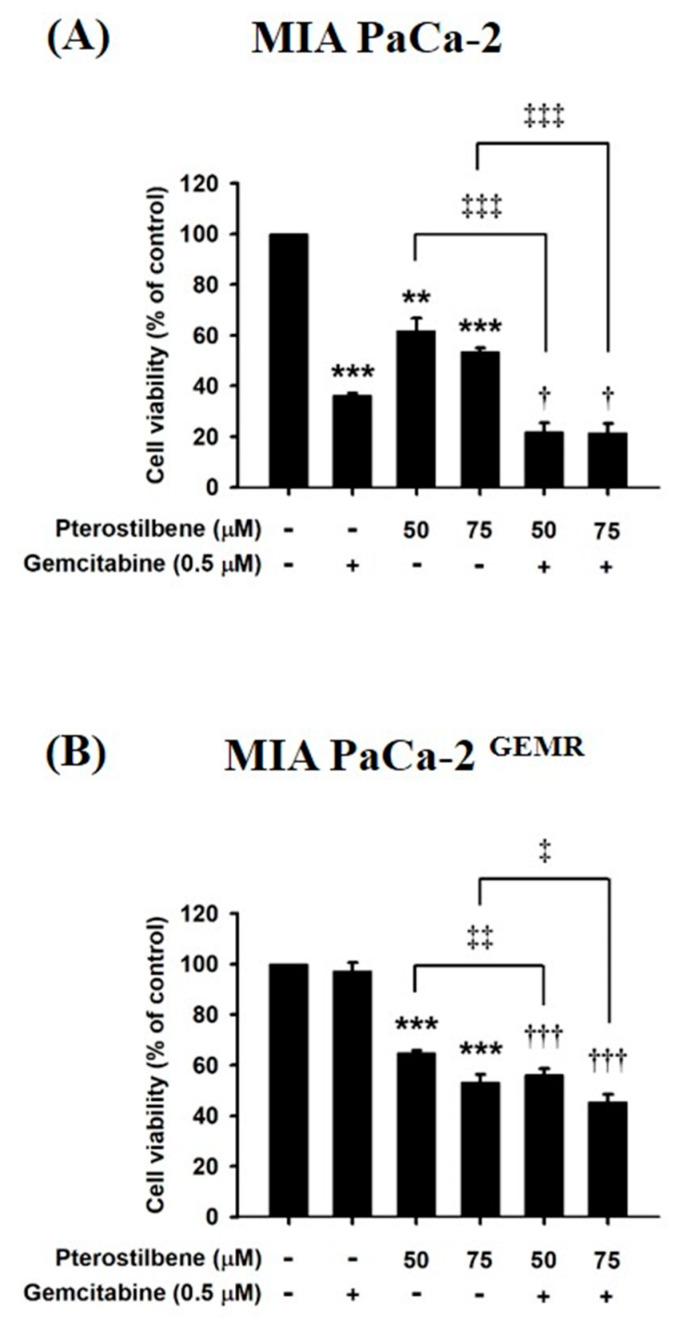
Effects of pterostilbene and gemcitabine cotreatment on MIA PaCa-2 and MIA PaCa-2 ^GEMR^ cell viability. Cells were treated with 50 or 75 μM pterostilbene combined with gemcitabine for 72 h. The cell viability of (**A**) MIA PaCa-2 and (**B**) MIA PaCa-2 ^GEMR^ cells was analyzed by MTT assay. The results are expressed as the mean ± SD (*n* = 3). *p* values were considered statistically significant when *p < 0.05, **p < 0.01, and ***p < 0.001 compared with untreated control. The values were considered statistically significant at †*p* < 0.05 and †††*p* < 0.001, compared with the gemcitabine group. The values were considered statistically significant at ‡*p* < 0.05, ‡‡*p* < 0.01 and ‡‡‡*p* < 0.001, compared with the pterostilbene group.

To examine whether chemosensitivity was enhanced by PTE incubation, the inhibitory effect of PTE combined with GEM on cell proliferation was assessed by MTT assay. In Figure 9A, treatment with 0.5 μM GEM dramatically prohibited cell proliferation in MIA PaCa-2 cells compared to untreated cells. Moreover, cell proliferation was inhibited 50 and 75 μM PTE-pretreated MIA PaCa-2 cells (Figure 9A). Not surprisingly, GEM treatment led to extensive cell death in 50 and 75 μM PTE-pretreated MIA PaCa-2 cells (Figure 9A). Remarkably, treatment with PTE combined with GEM significantly reduced cell viability compared with that in GEM-treated MIA PaCa-2 cells (Figure 9A). Chemosensitivity was also evaluated in PTE-pretreated GEM-resistant cells. As anticipated, the cell viability of MIA PaCa-2 ^GEMR^ cells was not influenced by GEM treatment (Figure 9B). Cell proliferation was inhibited in PTE-treated cells compared to untreated MIA PaCa-2 ^GEMR^ cells in a dose-dependent manner (Figure 9B). In addition, PTE combined with GEM notably enhanced MIA PaCa-2 ^GEMR^ cell cytotoxicity compared to that with GEM treatment alone or PTE pretreatment alone (Figure 9B).

## 4. Discussion

PDAC is a very aggressive form of cancer, and patients have a five-year survival rate lower than 6% because GEM resistance develops within weeks after chemotherapy initiation. Unfortunately, approximately 75% of PDAC patients have GEM resistance, which has a long-lasting effect on global disease burden [4]. Therefore, finding novel effective preventative strategies is urgently required. Cancer chemoprevention with dietary phytochemicals has been discovered because of their high antitumor capacity and limited side effects [27]. PTE, a bioactive component of various *Vaccinium* berries, has been shown to have a tumor suppression effect in colorectal cancer, lung cancer, and human melanomas [28,29,30]. Likewise, our previous reports demonstrated that PTE restricts breast cancer cell motility, invasion, and metastasis via modulation of the tumor microenvironment and epithelial-mesenchymal transition (EMT)-associated signaling pathways [14,31]. In the present study, we found that the chemopreventive effect of PTE occurs through inducing apoptosis and autophagy in both MIA PaCa-2 cells and MIA PaCa-2 ^GEMR^ cells.

Some studies indicate that cell cycle arrest in cancer cells can eventually induce apoptosis [32,33]. Enhancement of cell cycle arrest is a successful strategy for attenuating of cancer development and progression. Our results showed that 25–75 μM PTE exhibited a powerful cytotoxic effect that not only resulted in S-phase cell cycle arrest, but also increased apoptosis-associated protein expression in a dose-dependent manner in both PDAC cell lines. Consistently, other reports also found that PTE triggers S-phase cell cycle arrest and apoptosis in colon cancer cells and diffuse large B-cell lymphoma cells (DLBCL) [34,35]. Next, our research results were in agreement with previous reports demonstrating that PTE treatment induces autophagy and inhibits tumor progression in human bladder cancer and hepatocellular carcinoma cells [13,36]. For example, PTE treatment significantly increases autophagy-associated protein LC3-II and Bcl-xl (negative regulator of apoptosis) levels through AKT/mTOR/p70S6K axis regulation in T24 human bladder cancer cells [13]. In the present study, similar promoting effects on autophagy and apoptosis were observed in PTE-treated cells. However, another study showed that PTE inhibits human hepatocellular carcinoma cell growth by apoptosis-independent signaling [36]. Lifting endoplasmic reticulum (ER) stress accelerates PTE-induced autophagy in human hepatocellular carcinoma cells, indicating that tumor-specific cytotoxicity responses such as cell cycle arrest, ER stress, apoptosis, and autophagy may be elicited by PTE treatment.

Although PTE-induced antioxidant-, anti-inflammatory- and tumor cytotoxicity-related mechanisms have been established, the underlying mechanism of PTE-mediated chemosensitivity enhancement in PDAC is still unclear. A previous report showed that PTE induces apoptosis and autophagy in chemoresistant lung cancer cells and bladder cancer cells, which is initiated by the suppression of AKT signaling [13,15]. Additionally, PTE induces autophagy- and apoptosis-related cell death and inhibits MDR1 expression through Akt-Ser473 phosphorylation in cisplatin-resistant human oral cancer CAL 27 cells [37]. In agreement with this, our results showed that PTE triggered cell cycle arrest and apoptosis- and autophagy-mediated cytotoxicity and inhibited MDR1 expression by regulating the RAGE/PI3K/Akt axis. Although several natural compounds are used in combined therapies against pancreatic cancer such as catechins, resveratrol, and curcumin [38,39,40]. However, few available publications showed that natural compounds directly target MDR1 expression and promote chemosensitivity. Our results support the idea that PTE is an MDR1 modulator that increases GEM-induced tumor cell death in PDAC.

Gemcitabine (GEM) drug resistance causes high mortality rates and poor outcomes in PDAC patients. RAGE has been demonstrated to be involved in the GEM resistance process. Therefore, finding a safe and effective way to inhibit RAGE-initiated GEM resistance is urgent. Pterostilbene (PTE), a natural methoxylated analogue derived from resveratrol and found in grapes and blueberries, has diverse bioactivities such as antioxidative, anti-inflammatory, and anticancer characteristics. Based on our results, it was demonstrated that PTE induced S-phase cell cycle arrest and apoptotic and autophagic cell death and inhibited MDR1 expression by inhibiting the RAGE/PI3K/Akt axis, which consequently enhanced chemosensitivity in both MIA PaCa-2 cells. Remarkably, convincing evidence was established by RAGE small interfering RNA transfection.

## 5. Conclusions

Taken together, our study demonstrates that PTE promotes chemosensitivity by inhibiting cell proliferation and MDR1 expression via the RAGE/PI3K/Akt signaling pathway in PDAC cells. The observations in these experiments indicate that PTE may play a pivotal role in MDR1 modulation for PDAC chemoprevention.

## Abbreviations

Atg	autophagy gene
Bax	Bcl2-associated X Protein
Bcl-xL	B-cell lymphoma-extra large
GEM	Gemcitabine
LC3	microtubule-associated protein light chain 3
MDR1	multidrug resistance protein 1
PDAC	pancreatic ductal adenocarcinoma
PTE	Pterostilbene
RAGE	receptor for advanced glycation end products
siRNA	small interfering RNA

## References

[1] Kindler H.L. (2018). A glimmer of hope for pancreatic cancer. N. Engl. J. Med..

[2] Singhi A.D., Koay E.J., Chari S.T., Maitra A. (2019). Early detection of pancreatic cancer: Opportunities and challenges. Gastroenterology.

[3] Rahib L., Smith B.D., Aizenberg R., Rosenzweig A.B., Fleshman J.M., Matrisian L.M. (2014). Projecting cancer incidence and deaths to 2030: The unexpected burden of thyroid, liver, and pancreas cancers in the United States. Cancer Res..

[4] Binenbaum Y., Na’ara S., Gil Z. (2015). Gemcitabine resistance in pancreatic ductal adenocarcinoma. Drug Resist. Updates.

[5] Vaccaro G., Lopez C.D. (2017). Chemoradiation for locally advanced unresectable pancreatic cancer-what now?. JAMA Oncol..

[6] Hayashi T., Nakamura T., Kimura Y., Yoshida M., Someya M., Kawakami H., Sakuhara Y., Katoh N., Takahashi K., Ambo Y. (2019). Phase 2 study of neoadjuvant treatment of sequential s-1-based concurrent chemoradiation therapy followed by systemic chemotherapy with gemcitabine for borderline resectable pancreatic adenocarcinoma (HOPS-BR 01). Int. J. Radiat. Oncol. Biol. Phys..

[7] Tsai H.Y., Ho C.T., Chen Y.K. (2017). Biological actions and molecular effects of resveratrol, pterostilbene, and 3′-hydroxypterostilbene. J. Food Drug Anal..

[8] Tolomeo M., Grimaudo S., Di Cristina A., Roberti M., Pizzirani D., Meli M., Dusonchet L., Gebbia N., Abbadessa V., Crosta L. (2005). Pterostilbene and 3’-hydroxypterostilbene are effective apoptosis-inducing agents in MDR and BCR-ABL-expressing leukemia cells. Int. J. Biochem. Cell Biol..

[9] Schneider J.G., Alosi J.A., McDonald D.E., McFadden D.W. (2010). Pterostilbene inhibits lung cancer through induction of apoptosis. J. Surg. Res..

[10] Paul S., Rimando A.M., Lee H.J., Ji Y., Reddy B.S., Suh N. (2009). Anti-inflammatory action of pterostilbene is mediated through the p38 mitogen-activated protein kinase pathway in colon cancer cells. Cancer Prev. Res. (Phila).

[11] Pan M.H., Chang Y.H., Badmaev V., Nagabhushanam K., Ho C.T. (2007). Pterostilbene induces apoptosis and cell cycle arrest in human gastric carcinoma cells. J. Agric. Food Chem..

[12] Lin V.C., Tsai Y.C., Lin J.N., Fan L.L., Pan M.H., Ho C.T., Wu J.Y., Way T.D. (2012). Activation of AMPK by pterostilbene suppresses lipogenesis and cell-cycle progression in p53 positive and negative human prostate cancer cells. J. Agric. Food Chem..

[13] Chen R.J., Ho C.T., Wang Y.J. (2010). Pterostilbene induces autophagy and apoptosis in sensitive and chemoresistant human bladder cancer cells. Mol. Nutr. Food Res..

[14] Hong B.H., Wu C.H., Yeh C.T., Yen G.C. (2013). Invadopodia-associated proteins blockade as a novel mechanism for 6-shogaol and pterostilbene to reduce breast cancer cell motility and invasion. Mol. Nutr. Food Res..

[15] Hsieh M.J., Lin C.W., Yang S.F., Sheu G.T., Yu Y.Y., Chen M.K., Chiou H.L. (2014). A combination of pterostilbene with autophagy inhibitors exerts efficient apoptotic characteristics in both chemosensitive and chemoresistant lung cancer cells. Toxicol. Sci..

[16] Kang R., Tang D., Schapiro N.E., Loux T., Livesey K.M., Billiar T.R., Wang H., Van Houten B., Lotze M.T., Zeh H.J. (2014). The HMGB1/RAGE inflammatory pathway promotes pancreatic tumor growth by regulating mitochondrial bioenergetics. Oncogene.

[17] Lan C.Y., Chen S.Y., Kuo C.W., Lu C.C., Yen G.C. (2019). Quercetin facilitates cell death and chemosensitivity through RAGE/PI3K/AKT/mTOR axis in human pancreatic cancer cells. J. Food Drug Anal..

[18] Mikstacka R., Rimando A.M., Ignatowicz E. (2010). Antioxidant effect of trans-resveratrol, pterostilbene, quercetin and their combinations in human erythrocytes in vitro. Plant Foods Hum. Nutr..

[19] Yu W.Z., Hu X.Q., Wang M.F. (2018). Pterostilbene inhibited advanced glycation end products (AGEs)-induced oxidative stress and inflammation by regulation of RAGE/MAPK/NF-kappa B in RAW264.7 cells. J. Funct. Foods.

[20] Wen W., Lowe G., Roberts C.M., Finlay J., Han E.S., Glackin C.A., Dellinger T.H. (2018). Pterostilbene suppresses ovarian cancer growth via induction of apoptosis and blockade of cell cycle progression involving inhibition of the STAT3 pathway. Int. J. Mol. Sci..

[21] Mayer I.A., Arteaga C.L. (2016). The PI3K/AKT pathway as a target for cancer treatment. Annu. Rev. Med..

[22] Guerrero-Zotano A., Mayer I.A., Arteaga C.L. (2016). PI3K/AKT/mTOR: Role in breast cancer progression, drug resistance, and treatment. Cancer Metastasis Rev..

[23] Shahab U., Ahmad M.K., Mahdi A.A., Waseem M., Arif B., Moinuddin, Ahmad S. (2018). The receptor for advanced glycation end products: A fuel to pancreatic cancer. Semin. Cancer Biol..

[24] Bresnick A.R., Weber D.J., Zimmer D.B. (2015). S100 proteins in cancer. Nat. Rev. Cancer.

[25] Notte A., Leclere L., Michiels C. (2011). Autophagy as a mediator of chemotherapy-induced cell death in cancer. Biochem. Pharmacol..

[26] He L., Lai H., Chen T. (2015). Dual-function nanosystem for synergetic cancer chemo-/radiotherapy through ROS-mediated signaling pathways. Biomaterials.

[27] Surh Y.J. (2003). Cancer chemoprevention with dietary phytochemicals. Nat. Rev. Cancer.

[28] Jung J.H., Shin E.A., Kim J.H., Sim D.Y., Lee H., Park J.E., Lee H.J., Kim S.H. (2019). NEDD9 inhibition by miR-25-5p activation is critically involved in co-treatment of melatonin- and pterostilbene-induced apoptosis in colorectal cancer cells. Cancers (Basel).

[29] Chen R.J., Wu P.H., Ho C.T., Way T.D., Pan M.H., Chen H.M., Ho Y.S., Wang Y.J. (2017). P53-dependent downregulation of hTERT protein expression and telomerase activity induces senescence in lung cancer cells as a result of pterostilbene treatment. Cell Death Dis..

[30] Benlloch M., Obrador E., Valles S.L., Rodriguez M.L., Sirerol J.A., Alcacer J., Pellicer J.A., Salvador R., Cerda C., Saez G.T. (2016). Pterostilbene decreases the antioxidant defenses of aggressive cancer cells in vivo: A physiological glucocorticoids- and Nrf2-dependent mechanism. Antioxid. Redox Signal..

[31] Mak K.K., Wu A.T., Lee W.H., Chang T.C., Chiou J.F., Wang L.S., Wu C.H., Huang C.Y., Shieh Y.S., Chao T.Y. (2013). Pterostilbene, a bioactive component of blueberries, suppresses the generation of breast cancer stem cells within tumor microenvironment and metastasis via modulating NF-kappaB/microRNA 448 circuit. Mol. Nutr. Food Res..

[32] Xu Z., Zhang F., Bai C., Yao C., Zhong H., Zou C., Chen X. (2017). Sophoridine induces apoptosis and S phase arrest via ROS-dependent JNK and ERK activation in human pancreatic cancer cells. J. Exp. Clin. Cancer Res..

[33] de Carvalho N.C., Neves S.P., Dias R.B., Valverde L.F., Sales C.B.S., Rocha C.A.G., Soares M.B.P., Dos Santos E.R., Oliveira R.M.M., Carlos R.M. (2018). A novel ruthenium complex with xanthoxylin induces S-phase arrest and causes ERK1/2-mediated apoptosis in HepG2 cells through a p53-independent pathway. Cell Death Dis..

[34] Kong Y., Chen G., Xu Z., Yang G., Li B., Wu X., Xiao W., Xie B., Hu L., Sun X. (2016). Pterostilbene induces apoptosis and cell cycle arrest in diffuse large B-cell lymphoma cells. Sci. Rep..

[35] Chang G., Xiao W., Xu Z., Yu D., Li B., Zhang Y., Sun X., Xie Y., Chang S., Gao L. (2017). Pterostilbene induces cell apoptosis and cell cycle arrest in T-cell leukemia/lymphoma by suppressing the ERK1/2 pathway. Biomed. Res. Int..

[36] Yu C.L., Yang S.F., Hung T.W., Lin C.L., Hsieh Y.H., Chiou H.L. (2019). Inhibition of eIF2alpha dephosphorylation accelerates pterostilbene-induced cell death in human hepatocellular carcinoma cells in an ER stress and autophagy-dependent manner. Cell Death Dis..

[37] Chang H.P., Lu C.C., Chiang J.H., Tsai F.J., Juan Y.N., Tsao J.W., Chiu H.Y., Yang J.S. (2018). Pterostilbene modulates the suppression of multidrug resistance protein 1 and triggers autophagic and apoptotic mechanisms in cisplatin-resistant human oral cancer CAR cells via AKT signaling. Int. J. Oncol..

[38] Appari M., Babu K.R., Kaczorowski A., Gross W., Herr I. (2014). Sulforaphane, quercetin and catechins complement each other in elimination of advanced pancreatic cancer by miR-let-7 induction and K-ras inhibition. Int. J. Oncol..

[39] Michel O., Przystupski D., Saczko J., Szewczyk A., Niedzielska N., Rossowska J., Kulbacka J. (2018). The favourable effect of catechin in electrochemotherapy in human pancreatic cancer cells. Acta. Biochim. Pol..

[40] Lohse I., Wildermuth E., Brothers S.P. (2018). Naturally occurring compounds as pancreatic cancer therapeutics. Oncotarget.

